# Travoprost Induced Granulomatous Anterior Uveitis

**DOI:** 10.1155/2011/507073

**Published:** 2011-12-14

**Authors:** Patrick Chiam

**Affiliations:** Eye Care Centre, Leighton Hospital, Crewe, Cheshire CW1 4QJ, UK

## Abstract

*Purpose*. To report a case of granulomatous anterior uveitis caused by travoprost. *Methods*. Single observational case report. *Results*. A 71-year-old who was fit and healthy presented with bilateral granulomatous anterior uveitis 2 months after he was started on travoprost in both eyes. There was no past history of uveitis. Blood test and radiological investigation were unremarkable. Travoprost was stopped. The uveitis resolved on topical steroid treatment. A rechallenge with travoprost was attempted in one eye. The inflammation recurred in this eye only. This subsided with the cessation of travoprost alone without topical steroid. *Conclusion*. This is the first case report of travoprost causing granulomatous anterior uveitis. The uveitis recurred with a rechallenge. Changing the prostaglandin analogue to another topical treatment may be adequate to cease the inflammation.

## 1. Introduction

Prostaglandin analogues are well known to cause nongranulomatous anterior uveitis. However, bimatoprost and latanoprost have also been reported to cause granulomatous anterior uveitis. This is the first case report that the latter can be induced by travoprost as well.

## 2. Case Report

A 71-year-old Caucasian gentleman presented with blurry vision and uncomfortable eyes. His visual acuities were 6/24 in both eyes. He complained his visual had been deteriorating a fortnight ago. He was started on travoprost 2 months ago. The treatment was added to dorzolamide to improve the intraocular pressure (IOP) control. He had bilateral glaucomatous optic neuropathy, more marked in the left eye. The IOPs were 34 mmHg in the right eye and 45 mmHg in the left when he was seen in the eye emergency clinic.

He was usually fit and well. He suffered from poor hearing since 2 years ago. He was on lisinopril for hypertension and atorvastatin. He had been taking Systane for dysfunctional tear syndrome twice to four times daily over the past 2 years. There were no other symptoms or signs to elicit.

There were mutton-fat keratic precipitates and 3+ cells in the anterior chambers with posterior synechiae. Dilated fundoscopy revealed mild vitritis but no signs of posterior uveitis. The patient was started on intense topical steroid and mydrilate. The travoprost was switched to a fixed combination of brinzolamide and timolol. Oral acetazolamide was prescribed.

The eyes were quieter after 3 days, and the IOPs were 23 mmHg in both eyes. A week later, the eyes were quiet, and there were less mutton-fat keratic precipitates. The IOPs were 12 and 14 mmHg. He was advised to stop the acetazolamide.

The inflammatory markers were normal. The haemoglobin was slightly below normal at 12.5 g/dL, however, the rest of the full blood count was normal. The results for immunological screen, inflammatory markers, syphilis, tuberculosis, toxoplasma, Lyme disease, angiotensin converting enzyme, and calcium profile were unremarkable. The chest X-ray was normal.

A month from the initial presentation, his eyes were quiet on topical steroid three times daily. The IOPs were 17 and 18 mmHg on topical brinzolamide and timolol. The possibility of the granulomatous anterior uveitis secondary to travoprost was considered. The steroid was stopped. The patient agreed to a rechallenge with travoprost in the right eye only. Both eyes were quiet in the first week; however, the right eye developed 2+ cells in the anterior chamber a fortnight later. The left eye remained quiet. Travoprost was stopped. In the subsequent visits, the uveitis resolved without any topical steroid. The patient's eyes had been quiet over the past 3 months on the fixed brinzolamide and timolol combination topical treatment.

## 3. Conclusion

Prostaglandin analogues are well known to cause nongranulomatous anterior uveitis. However, only a handful of granulomatous anterior uveitis cases have been reported for latanoprost [1] and bimatoprost [2]. To date, there has been no similar report in the literature for travoprost. However, the latter has been implicated in nongranulomatous anterior uveitis [3–5].

The mechanism of induction of intraocular inflammation by prostaglandin analogue has not been entirely clear. It has been proposed that prostaglandin F_2*α*_ causes the release of prostaglandin E_2_ [6]. This in turn stimulates the release of arachidonic acid by activating phospholipase II [7]. Arachidonic acid promotes the production of eicosanoids and other proinflammatory mediators in the eye, resulting in changes in the blood aqueous barriers.

The improvement of the inflammation with the cessation of travoprost and its recurrence after the rechallenge of the medication suggest a causal relationship. Based on the Naranjo algorithm [8] for determining the cause of an adverse drug reaction, the score for this patient is 9, which suggests a definite link between the use of travoprost and granulomatous anterior uveitis (score of 5–8 implies a probable link and 9 or more indicate a definite link). Although there is a possibility that the patient may be allergic to the preservative component in travoprost (Polyquad), this remains unlikely since he had used Systane in the past without any side effect. The artificial tear also contains the same preservative found in travoprost. This is the first case report to describe granulomatous anterior uveitis caused by travoprost.
